# First Evidence of the Presence of Anatoxin-A in Sea Figs Associated with Human Food Poisonings in France

**DOI:** 10.3390/md18060285

**Published:** 2020-05-29

**Authors:** Ronel Biré, Thomas Bertin, Inès Dom, Vincent Hort, Corinne Schmitt, Jorge Diogène, Rodolphe Lemée, Luc De Haro, Marina Nicolas

**Affiliations:** 1 Laboratory for Food Safety, Université Paris-Est, ANSES, F-94701 Maisons-Alfort, France; bertin.thomas94@gmail.com (T.B.); ines.dom.tn@gmail.com (I.D.); vincent.hort@anses.fr (V.H.); marina.nicolas@anses.fr (M.N.); 2 Clinical Pharmacology, Poison Control Center, St Marguerite Hospital, 13009 Marseille, France; corinne.schmitt@ap-hm.fr (C.S.); luc.deharo@ap-hm.fr (L.D.H.); 3 Marine Continental Waters, IRTA, Ctra. Poble Nou, km 5.5, 43540 Sant Carles de la Ràpita, Spain; jorge.diogene@irta.cat; 4 Laboratoire d’Océanographie de Villefranche, Sorbonne Université, CNRS, LOV, F-06230 Villefranche-sur-Mer, France; lemee@obs-vlfr.fr

**Keywords:** sea figs, *Microcosmus*, human food poisoning, cyanotoxins, anatoxin-a

## Abstract

From January 2011 to March 2018, 26 patients aged from 20 to 80 years old reported being sick in France after eating sea figs of the genus *Microcosmus*. The patients had symptoms evoking a cerebellar syndrome: blurred or double vision, ataxia and dizziness, asthenia, headache, muscle cramps, paresthesia and digestive disorders (nausea, vomiting and diarrhea). Three of the 18 food poisoning events recorded by the Poison Control Center in Marseille and involving four patients were further investigated as the meal leftovers were collected and analyzed. A previous study ruled out the presence of the regulated lipophilic marine toxins after high-resolution mass spectrometry, but further analyses were required to look for hydrophilic cyanotoxins. The sea fig leftovers from food poisoning case Numbers 1 (January 2011), 6 (December 2012) and 17 (March 2018) of this published case series were analyzed by hydrophilic interaction liquid chromatography coupled to low- and high-resolution mass spectrometry to investigate the presence of hydrophilic cyanotoxins. The sea fig samples showed anatoxin-a (ATX-a) concentrations ranging from 193.7 to 1240.2 µg/kg. The sea fig control sample analyzed was also contaminated with ATX-a but in a much smaller concentration (22.5 µg/kg). To the best of our knowledge, this is the first report of human food poisoning involving ATX-a as the possible causative toxin where the cyanotoxin could be unequivocally identified.

## 1. Introduction

Sea figs, also called violets, sea squirts, sea lemons or sea potatoes, depending on the country, are marine organisms (Tunicates) belonging to the class of the Ascidians. They are eaten along the French Mediterranean coast and are considered as a delicacy by consumers fond of their strong iodine taste. They feed by filtering the seawater, and like other filter-feeding organisms, are likely to accumulate contaminants present in the water column. There is a report in the literature on the contamination of sea figs of the genus *Microcosmus* with saxitoxins (STXs) [1]. Others Tunicates were also found to be contaminated with marine biotoxins such as STXs and tetrodotoxin [2] or domoic acid (DA) [3].

When investigating the contamination of marine organisms by natural toxins, the first lead naturally points towards marine toxins. However, there are reports in the literature of the presence of cyanotoxins in the marine environment [4,5]. This phenomenon is likely to take place in estuarine environments with cyanobateria migrating from freshwater to marine environments. Another explanation for the presence of freshwater toxins into the marine waters can come from the toxin producer itself. Thus, marine cyanobacteria belonging to the genus *Hydrocoleum* were identified as the producer of anatoxins (ATXs) [6,7]. A review on ATXs reported the occurrence of these cyanotoxins in water and in biota, including fish, dogs, Flamingos and in food supplements [8]. ATXs are secondary amine bicyclic alkaloids. Different techniques were used for their analysis in various matrices: this included liquid chromatography (LC) coupled with ultra-violet (UV) detection [9] or with fluorescence detection after a derivatization step using 4-fluoro-7-nitro-2,1,3-benzoxadiazole to make the toxins fluorescent [10,11,12,13]. The use of gas chromatography coupled with mass chromatography also requires a derivatization procedure to transform ATX-a into a more volatile n-acetyl derivative [13]. LC coupled with mass spectrometry (MS) is widely used for the analysis of ATXs in a variety of matrices, including cyanobacteria, animals, vegetables and food supplements [8,14]. One of the major problems associated with the analysis of ATXs is related to the fact that ATX-a can be misidentified with the amino acid phenylalanine (Phe), its isobaric counterpart. In single MS, both compounds can be mistaken, unless they are chromatographically separated. This is difficult to achieve in reversed phase liquid chromatography due to the polar nature of ATX-a and Phe, but not impossible [15]. A methylation step can help remove the Phe signal to further ascertain the identity of ATX-a [15]. The use of hydrophilic interaction liquid chromatography (HILIC) is dedicated to the separation of polar compounds, including ATXs [16]. To unequivocally distinguish ATX-a from Phe it is possible to couple the separation capabilities of HILIC with the resolving power of high-resolution mass spectrometry (HRMS), as both compounds have different masses (ATX-a: MH^+^ m/z 166.1226; Phe: MH^+^ *m*/*z* 166.0862).

From January 2011 to March 2018, the Poison Control Center in Marseille (PCCM) registered 18 food poisoning events associated with the consumption of sea figs belonging to the *Microcosmus* genus [17]. The 26 patients involved (16 women and 10 men) aged 20 to 80 years old, reported a series of symptoms evoking a cerebellar syndrome. The most reported symptoms included ataxia, dizziness and accommodation disturbances (blurred vision, diplopia). Digestive disorders (nausea, vomiting and diarrhea) were also reported but were less frequent; so were asthenia, headaches, muscle cramps and paresthesia. The onset of the symptoms started 10 to 90 min following the ingestion of the sea figs. All the patients recovered completely in a timeframe going from one to 30 h.

Meal leftovers collected from the patients involved in case Numbers 1 (January 2011, 2 men, 30 and 52 years old) and 6 (December 2012, 1 man, 60 years old) of the published case series were sent to both the French National Reference Laboratory for Marine Biotoxins (NRLMB) and to the Institute of Agrifood Research and Technology (IRTA) in Spain. The analyses performed at IRTA excluded the presence of the regulated marine biotoxins: STXs, DA and its isomers, and the lipophilic toxins (okadaic acid, dinophysistoxins, azaspiracids, yessotoxins and pectenotoxins). Yet, a hemolytic activity was detected, although different from the one elicited by palytoxin (J. Diogène, personal communication). The meal leftovers from the patient of case Number 17 (March 2018, 1 woman, 63 years old) were sent to the French NRLMB and analyzed along with the leftovers of cases 1 and 6, using liquid chromatography coupled to high-resolution mass spectrometry (LC-HRMS) using the non-target analysis method developed by Dom et al. [18]. The suspect screening analysis performed on a list of 821 toxins, including marine toxins and cyanotoxins, was not conclusive regarding the nature of the potential contaminants present in the sea fig-contaminated samples, by comparison with a reference sample. The presence of ATX-a was suspected in one of the contaminated samples, on the basis of the in silico fragmentation pattern, but was ruled out because the feature corresponding to ATX-a was only found in one of the triplicate injections [19]. Furthermore, the analytical conditions used were optimized for lipophilic compounds and consequently were not appropriate for hydrophilic toxins. In these conditions, ATX-a and its isobaric counterpart, Phe, an amino acid frequently encountered in biological matrices, coelute, making it difficult to distinguish them.

In this article, we describe the work carried out to further investigate the presence of hydrophilic toxins and to unequivocally identify ATX-a in the sea figs associated with human food poisoning in France, by hydrophilic interaction liquid chromatography (HILIC) coupled to mass spectrometry, both in low- (LR) and high-resolution (HR).

## 2. Results

### 2.1. HILIC-MS Analysis in Low Resolution

**Figure 1:**
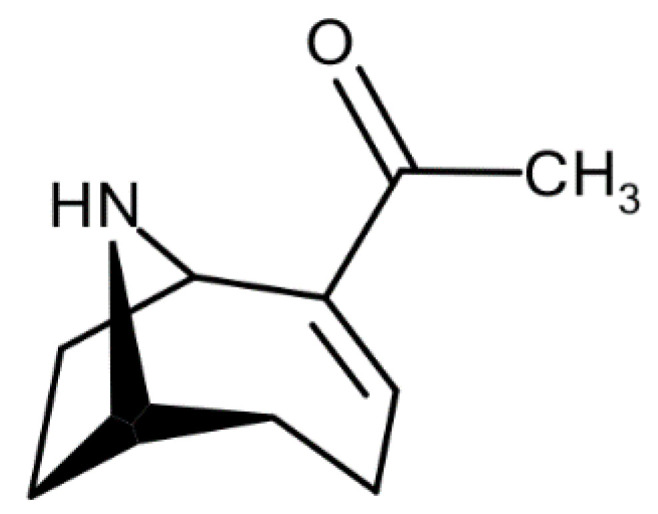
Structure of ATX-a.

**Figure 2:**
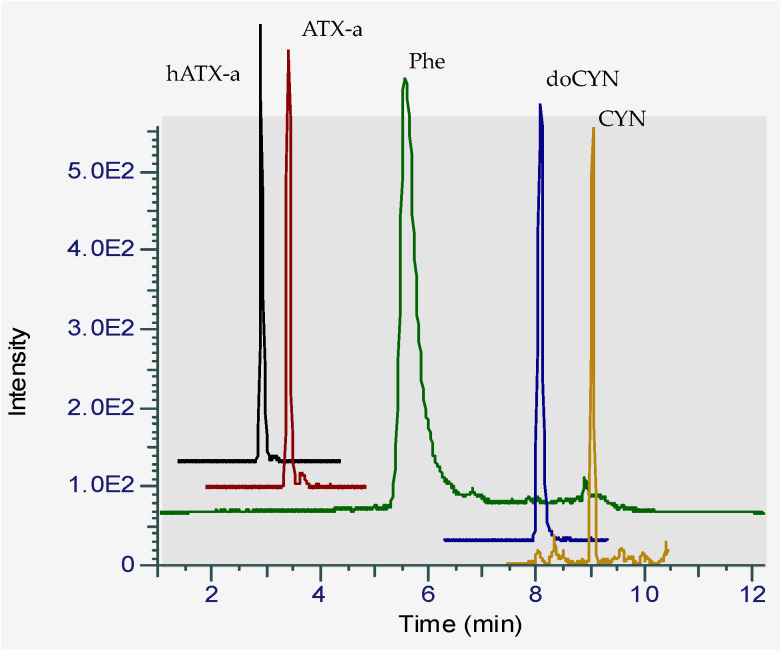
Chromatogram of a standard mixture of four hydrophilic cyanotoxins in solvent analyzed by HILIC-LRMS showing the separation of ATX-a and Phe in the chromatographic conditions adapted from Turner et al. [20].

A HILIC-LRMS method, adapted from Turner et al. [20], so as to determine the hydrophilic cyanotoxins, ATX-a (Figure 1), hATX-a, cylindrospermopsin (CYN) and deoxy-cylindrospermopsin (doCYN) further to saxitoxins (STXs) and tetrodotoxin (TTX), was implemented by the French NRLMB as part of the national scheme for monitoring emerging toxins. The method had been validated for ATX-a, STXs, TTX, CYN and doCYN in mussels and oysters in the frame of the national monitoring scheme and was applied to sea fig samples in this study. The extraction efficiency of the method determined for ATX-a at two concentration levels (17.5 and 35 µg/kg) in mussels and oysters ranged from 93% to 95% (data not shown). Most importantly, the method enables the separation of the isobaric compounds, ATX-a and Phe (Figure 2).

A series of quality criteria (QCs) were monitored to ensure that the toxins sought in the samples were correctly identified in HILIC-LRMS. The QCs included the shift in retention time between the ATX-a peak in the standard and in the samples (acceptance value ≤ 5%) and the intensity ratio of the ATX-a qualitative and quantitative transitions (18% ≤ acceptance value ≤ 33%). From the selectivity point of view, crucial criteria in HILIC-LRMS consists in making sure that the chromatographic conditions enable a baseline separation of ATX-a and its isobaric counterpart, Phe. All criteria met the requirements set, as the shift in retention time was 1.4% maximum for all samples analyzed in the same run, ATX-a and Phe eluted several minutes apart and the ion ratio was in the accepted range (18–33%).

**Figure 3:**
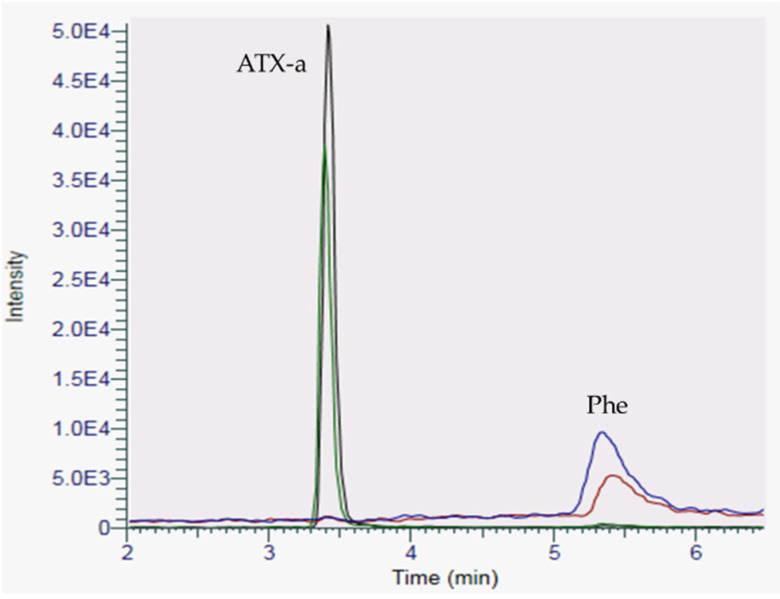
Chromatogram of the sea fig sample FP-17-2018, before (green trace for ATX-a and blue trace for Phe) and after spiking with ATX-a (black trace for ATX-a and red trace for Phe) using HILIC-LRMS analysis.

To further confirm that the chromatographic peak detected in the sea fig samples was correctly attributed to ATX-a, sample FP-17-2018 involved in the food poisoning case 17 was spiked with a standard of ATX-a. As shown in Figure 3, after spiking, there is a unique peak corresponding to ATX-a and the increase in intensity of the peak (+43%) is correlated with the amount of ATX-a standard added to the sample extract.

To ascertain the accuracy of the toxin concentration in the sea fig samples, the matrix effects were determined in the control sample by comparing a calibration curve in solvent (acetonitrile 82.5% + acetic acid 0.25%) and in the aforementioned matrix (toxin spiked in the extract). A signal suppression of 18% was observed in the sea fig control sample compared to the calibration curve in solvent, so the matrix effects were considered as negligible (<±20%).

The analysis of the sea figs associated with the food poisoning case Numbers 1, 6 and 17, showed the presence of ATX-a in concentrations ranging from 193.7 to 1240.2 µg/kg (Table 1). Surprisingly, traces of ATX-a were also found in a sea fig sample bought on the market and analyzed as a negative control, whereas a mussel control sample was ATX-a-free (concentration below the detection limit). Yet, the toxin concentration in the sea fig control sample (22.5 µg/kg) was much lower than in the samples involved in the food poisoning events.

None of the other hydrophilic cyanotoxins monitored (hATX, CYN, doCYN) were detected in the samples. As for STXs, they were all below limit of detection.

### 2.2. Confirmatory Analysis Using HILIC-HRMS

To unequivocally confirm the presence of ATX-a in the sea figs, the samples were analyzed in HILIC-HRMS on a Sciex 5600 QTOF. The QCs we set in HRMS for the annotation of a peak are as follows: retention time (RT error <2%), mass error <5 ppm for the parent ion, isotope ratio difference <10%, purity score (matching of MS/MS fragmentation spectrum with that of a reference standard) >65%. All the above-mentioned QCs were met, which unequivocally confirmed the presence of ATX-a in the three samples associated with the human poisoning cases observed in 2011, 2012 and 2018, but also in the sea fig control sample.

**Figure 4:**
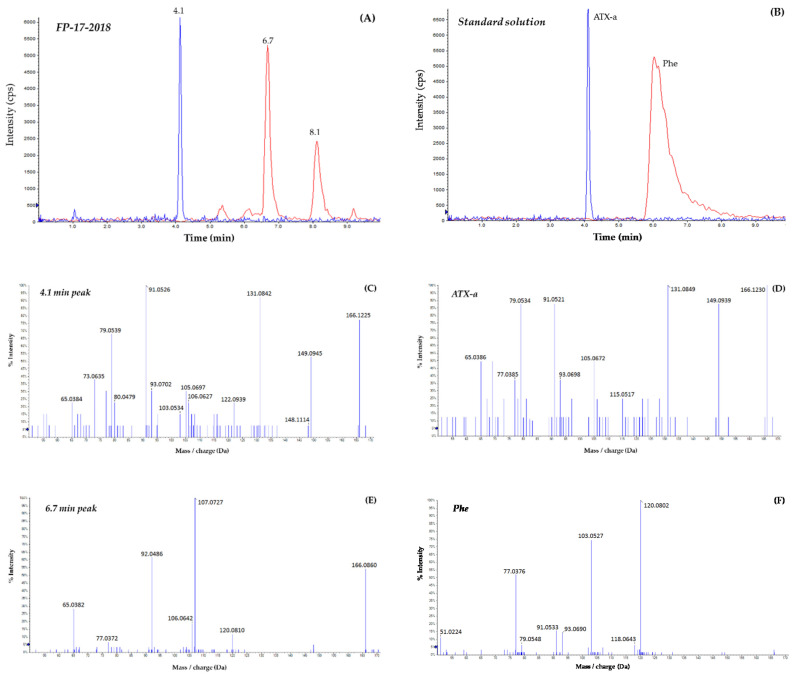
Extracted ion chromatograms and the corresponding MS/MS mass spectra of the sea fig sample FP-17-2018 (**A**,**C**,**E**) and a standard mixture of ATX-a and Phe (**B**,**D**,**F**). The sample extract and the standard solution were analyzed by HILIC-HRMS on a Sciex 5600 QTOF.

The sea fig control sample and the three sea fig samples involved in food poisoning events were analyzed by HILIC-HRMS in suspect screening mode. A list of 15 suspects was searched for (Table 2). This included ATX-a, hATX-a and their epoxy-, dihydro-, carboxy- and carboxy-dihydro-analogues. ATX-a was the only cyanotoxin found in the sea fig sample FP-17-2018 (Figure 4) and the other three samples (including the control). Indeed, all four samples showed a peak eluting at 4.1 min having the same retention time and MS/MS spectrum as the ATX-a standard analyzed in the same conditions (Figure 4B,D). The five major fragments specific to ATX-a and found in the sea figs samples were as follows: *m*/*z* 79.0539, 91.0526, 105.0697, 131.0842 and 149.0945. The ion trace corresponding to Phe showed two peaks eluting respectively at 6.7 and 8.1 min in the sea fig samples FP-01-2011, FP-06-2012, FP-17-2018 (Figure 4A) and in the control sample. The 6.7 min peak was the only one having a mass error <5 ppm compared to that of Phe. However, there was a slight shift in retention time compared to the Phe standard that eluted at 6.1 min (Figure 4B). Furthermore, the MS/MS spectra were not identical, as shown in Figure 4E,F. This raises questions about the identity of the compounds eluting at 6.7 and 8.1 min.

## 3. Discussion

This is the first time ATX-a has been reported in sea figs of the *Microcosmus* genus. This cyanotoxin was quantified in the meal leftovers of three food poisoning cases in France but might also be involved in the other 15 events of the published case series reported in France from 2011 to 2018, as the patients experienced a cerebellar syndrome with similar symptoms [17]. The presence of a freshwater cyanotoxin in a marine organism was unexpected but is most likely related to the filter-feeding behavior of the sea figs. The suspended materials (plankton, algal spores, bacteria and stirred-up detritus) enter with the water through the incurrent siphon, and they are caught in the pharynx and transported to the digestive tract [1]. There are reports in the literature of the presence of cyanotoxins in marine environment. Peacock et al. [5] reported the presence of microcystins (MCs) concomitantly with marine toxins in environmental samples (water and mussels) collected in the San Francisco Bay. Gibble et al. [4] examined the contamination and depuration of mussels and oysters experimentally exposed to MCs produced by the freshwater cyanobacterial genus *Microcystis*, showing the emerging threat related to this source of contamination of marine organisms. The migration of cyanobacterial cells from freshwater inflows to estuarine environment was demonstrated in Puget Sound (Washington), with the accumulation of MCs in mussels (*Mytilus trossulus*) [21]. Nevertheless, the hypothesis of cyanobacterial cells and/or toxins migrating from freshwater to marine environments could hardly explain the high concentrations in ATX-a found in the sea figs. This could be due to the presence of marine cyanobacteria, especially *Hydrocoleum lyngbyaceum* and *H. glutinosum*, as they are thought to produce potent toxins, including anatoxin-like, saxitoxin-like and ciguatoxin-like toxins, that can accumulate in marine organisms [6]. hATX-a was identified in mats of the benthic cyanobacterium *Hydrocoleum lyngbyaceum* and in the giant clam *Tridacna maxima*, associated with food poisoning events in New Caledonia [7]. The environmental survey conducted in New Caledonia in relation with ciguatera fish poisoning-like outbreaks focused on sampling areas across the intertidal and subtidal zones that are apparently conducive to the growth of marine cyanobacteria [6]. No information was available about the precise origin (exact FAO zone, described according to the Food and Agriculture Organization of the United States) and the habitat of the sea figs that were involved in the food poisoning events reported in France; therefore, it is not possible to tell whether this habitat would be suitable for marine cyanobacteria or not. Cyanobacteria of the genus *Hydrocoleum* and sea figs have in common the fact that they are both marine benthic organisms that can attach and live on hard surfaces; therefore, they are likely to coexist in the same habitat. To address this question, it would be necessary to sample the Mediterranean FAO zones where sea figs are generally harvested to collect Tunicates and marine cyanobacteria and to determine their toxin profiles for comparison purposes. 

There is no doubt about the identification of ATX-a in the sea figs, according to the retention time, the accurate mass and the mass spectra (fragmentation patterns). The chromatographic improvements made to the HILIC-LRMS method developed by Turner et al. [20] for the analysis of STXs and TTXs, enabled the analysis of different hydrophilic cyanotoxins (ATXs and CYNs) and the separation of ATX-a and Phe. These isobaric compounds could not be distinguished otherwise in LRMS, as the mass resolution is not good enough (ATX-a, *m*/*z* 166.1; Phe, *m*/*z* 166.1). The method was validated for ATX-a, notably on mussels and oysters, and was applied to sea fig samples. The experimental conditions enabled the identification of ATX-a in the sea fig samples involved in the food poisoning case Numbers 1, 6 and 17. The QCs implemented in LRMS helped strengthen the confidence in the identification of ATX-a, and the presence of this cyanotoxin in the sea figs was ultimately confirmed by HILIC-HRMS. Furey et al. [15] reported the use of HRMS to avoid the misidentification of ATX-a in reversed phase chromatography. The fragmentation patterns of ATX-a and Phe are identical to those reported in this paper. Furey et al. [15] mentioned six fragments identified as being related to ATX-a (*m*/*z* 79.0549, 91.0567, 107.0872, 131.0849 and 149.0943) and three for Phe (*m*/*z* 103.0530, 120.0803 and 131.0482) and they structurally identified all of them. If there was no doubt about the presence of ATX-a in the sea fig samples, two peaks were observed when monitoring the ion trace of Phe. The information gathered on both peaks indicates that none of them corresponds to Phe. An extensive list of ATX analogues, including the newly reported carboxy-ATX-a, carboxy-hATX-a and carboxy-dihydro ATX-a [22], was searched for in the sea figs by HILIC-HRMS (suspect screening mode). None of these analogues were detected but one cannot rule out the fact that unknown analogues may be present in the sea fig samples and may have gone undetected.

The association of ATX-a and/or hATX-a with dog neurotoxicosis has been reported in different countries [11,23,24,25,26,27,28,29,30,31]. The presence of ATX-a and microcystins (MCs) has been associated with Lesser Flamingos death in Kenya [32,33], but to the best of our knowledge, there is only one account in the literature about a human intoxication related to ATX-a and the identity of the toxin is controversial. In July 2002, a 17-year old boy died in Wisconsin, two days after swallowing water while swimming in a pond covered with scum. The stool and blood samples collected from the 17-year old boy and from another teenager who survived after exposure to the scum, revealed the presence of *Anabaena flos-aquae* (now *Dolichospermum flos-aquae*) and ATX-a [34]. Based on the available information, the coroner attributed the death to ATX-a, but these conclusions were subsequently questioned by Carmichael et al. as the time period between exposure and death (ca. 48 h) did not match the known mechanisms of toxicity of ATX-a. These authors hypothesized that the compound identified in the biological samples was Phe instead of ATX-a [35]. To the best of our knowledge, there is no other report in the literature of human poisoning possibly related to ATX-a, making our study the only one unequivocally associating the presence of ATX-a with human food poisoning cases.

As mentioned above, because of their filter-feeding behavior, the sea figs are likely to accumulate toxins as shellfish do. Roje Busatto et al. [1] reported the presence of STXs associated with human food poisoning in Croatia following the ingestion of *Microcosmus vulgaris*. The highest toxin level found in the sea figs sampled along the Croatian coast (eastern part of the Adriatic Sea) at the time of the food poisoning event was of 806.95 µg STX equivalent (eq.)/kg, that is just at the regulatory limit in shellfish (800 µg STX eq./kg). Yet, the symptoms reported (dizziness, vomiting, weakness in the legs and blurred vision) were not typical from STXs and more resembled a cerebellar syndrome. The typical symptoms associated with STXs and reported after food poisoning events generally include paresthesia of the extremities (fingers, toes) and the mouth, tingling sensation, numbness of the limbs, and respiratory difficulties, leading, in the severe cases, to the death of the patient without any breathing assistance [36]. Interestingly, the symptoms reported by Roje Busatto et al. resemble those observed in the French case series [17] and potentially associated with ATX-a. Retrospectively, it would be worth checking if STXs and ATX-a may not be present concomitantly in the sea figs responsible for the human food poisonings in Croatia.

ATX-a concentrations (193.7 to 1240.2 µg/kg) found in the sea fig leftovers in France are well above those reported in aquatic organisms, with a maximum of 36 µg/kg in fish muscle [8,14]. The ATX-a levels in dogs that died from neurotoxicosis are generally much higher than those we reported in the sea figs. The analysis of the stomach content of dogs that died in the Netherlands and in Germany after being exposed to cyanobacteria showed ATX-a concentrations up to 9500 ppb [25,26]. However, in some dog neurotoxicoses reported in the Germany and in the US, the ATX-a levels were a thousand times lower and in the range of 5–10 ppb [26,28]. ATX-a was also involved in the death of Lesser Flamingos in Africa, along with MCs, as evidenced by the analysis of the birds’ stomach content (4340 µg ATX-a/kg; [32]) and feathers (800 µg ATX-a/kg; [33]). In terms of occurrence, the data reported show that the ATX-a concentrations can greatly vary in the analyzed organisms, whatever their origin (aquatic or terrestrial). Toxin levels that intoxicated humans and killed animals cannot be compared per se, as we are dealing with very different species, different endpoints and different analytical conditions. However, one would expect the ATX-a concentrations that killed dogs and Flamingos to be higher than those possibly responsible for human food poisoning.

ATX-a is susceptible to photolysis, chemically instable and readily degraded via the digestive process, which probably limits the accumulation into aquatic organisms and explains why the occurrence data is sparse [8]. It is noteworthy that even sea figs bought on the market and intended to be used as a control sample were contaminated with ATX-a, although at much smaller levels than the meal leftovers from the food poisoning cases. A mussel control sample had to be used to have a real negative control. Additional sea fig controls collected from the market have to be analyzed to determine if there is an ATX-a background level in sea figs.

ATX-a is not regulated, and the World Health Organization could not derive a tolerable daily intake based on the scarce data available in the literature. Testai et al [14] proposed a provisional safe chronic reference value of 30 µg/day for an adult of 60 kg body weight. Additional data is required to derive appropriate health-based reference values. In the meantime, recommendations and/or a guidance value (GV) have been set in the US and Canada to protect human consumers. Thus, recommendations have been set in the US (Oregon, Washington State) and in Canada (Alberta) to reduce human exposure to cyanotoxins by limiting fish consumption and/or by removing the organs likely to accumulate cyanotoxins (viscera, fat) [37,38,39]. A threshold of 1100 µg/kg in fish was set in California [40]. If this GV was transposed to Tunicates, the sea figs involved in food poisoning case Numbers 6 and 17 (1240.2 and 1132.6 µg/kg, respectively) would be considered as unsafe but not the sea figs from case Number 1 (193.7 µg/kg). This raises questions about the toxin level required to make someone sick. The amount of food ingested by the patients is also necessary to evaluate the quantity of toxin the patient has been exposed to; unfortunately, this information is rarely available. The big difference in ATX-a concentration between case Number 1 and the other two cases is more likely related to the heterogeneity of contamination of individual sea figs. We cannot rule out the fact that the sea figs ingested by the two patients of case Number 1 might have been more contaminated than the analyzed meal leftovers. Another hypothesis would be that unknown ATX analogues of toxicological significance may be present in the sea figs involved in the food poisoning case Number 1 and not in the other sea fig samples.

## 4. Materials and Methods

### 4.1. Chemicals and Reagents

All solutions were prepared with analytical reagent-grade chemicals and ultrapure water (18.2 MΩ cm) obtained by purifying distilled water with the Milli-Q system associated with an Elix 5 purification system (Millipore S.A., St Quentin-en-Yvelines, France). LC-MS grade acetonitrile (MeCN), methanol (MeOH), glacial acetic acid (HAc), 98–100% formic acid (FA) and ammonium hydroxide solution (NH_4_OH, 25% as NH_3_) were purchased from Fisher Scientific (Loughborough, UK). STXs, ATX-a and CYN Certified reference standards were obtained from the National Research Council of Canada (NRC, Halifax, Nova Scotia, Canada). hATX and doCYN non-certified standards were purchased from Novakits (Nantes, France). 99% (S)-Phe of analytical grade was obtained from Merck (Darmstadt, Germany).

### 4.2. Sea Figs

Sea figs were collected from the patients involved in the food poisoning case Numbers 1 (January 2011), 6 (December 2012) and 17 (March 2018) of this published case series [17]. The sea figs were rinsed under tap water and were opened using a scalpel. The tissue was removed from the shell and homogenized using a blender prior to the analysis.

### 4.3. Extraction

The extraction procedure was adapted from Turner et al. [20], with several modifications. A double extraction was performed on sea fig samples. Typically, 1 ± 0.1 g of tissue homogenate was weighed into a centrifuge tube followed by the addition of 5.0 mL of 1% HAc, and the mixture was agitated for 90 s using a vortex-mixer. The samples were then placed into a boiling water bath for 5 min and were subsequently cooled down for 5 min in a cold-water bath under constant stirring. After a 90 s vortex-mixing step, the samples were centrifuged at 9000 rpm for 10 min. The supernatants were placed in another centrifuge tube and 5.0 mL of 1% Hac were added to the pellet. The samples were re-extracted by vortex-mixing for 90 s and then centrifuged at 9000 rpm for 10 min. Both supernatants were combined, and the volume was adjusted to 10.0 mL with 1% HAc. A 4.0 ml aliquot of sample was then neutralized with 20 µL of 25% ammonia and centrifuged at 15,000 rpm for 1 min.

### 4.4. Cleanup

The extracts were cleaned by solid phase extraction (SPE) using Supelclean ENVI-Carb 250 mg/3 mL SPE cartridges (Sigma-Aldrich, St. Louis, MO). The graphitized carbon cartridges were conditioned with 3 mL of a mixture of 30% MeCN and 1% HAc followed by 3 mL of 0.025% ammonia. An 800 µL volume of sample extract was loaded onto the SPE cartridge, which was subsequently washed with 700 µL of Milli-Q water. The toxins were eluted from the cartridge with 2 mL of a mixture of 30% MeCN and 1% HAc. SPE eluents were vortex-mixed and diluted to 1:4 with MeCN. The extracts were filtered on 0.22 µm Nylon filter (Macherey Nagel, Hoerdt, France) before HILIC-LCMS analysis in low- and high-resolution.

### 4.5. HILIC-MS Analysis

#### 4.5.1. HILIC-LRMS Analysis

The hydrophilic cyanotoxins, including the ATXs, were analyzed in HILIC-LRMS and the chromatographic conditions are described in Table 3 and Tables S1–S4 (Supplementary Materials). The chromatographic column used for the separation of the toxins (Table 3) was first conditioned using a cycle composed of three different chromatographic methods: (1) Conditioning (Supplementary Table S1), (2) Shutdown (Supplementary Table S2) and (3) Start-Up (Supplementary Table S3). The samples were then analyzed using the chromatographic separation conditions described in Supplementary Table S4. At the end of each sequence, the chromatographic system was post-conditioned using a cycle composed of two different chromatographic methods: (1) Conditioning (Supplementary Table S1) and (2) Shutdown (Supplementary Table S2).

The composition of the mobile phases used for the conditioning (pre- and post-sequence) and the analysis is as follows:

(A) H_2_O + 0.015% FA + 0.015% NH_3_
(B) 90% ACN + 0.01% FA
(C) H_2_O + 0.5% FA
(D) 100% MeOH

The same conditions were applied for the analysis of STXs by HILIC-LRMS, except that mobile phase B1 replaced mobile phase B2 (Supplementary Table S5).

LRMS analysis was performed with a TSQ Vantage triple quadrupole mass spectrometer (Thermo Fisher Scientific, San Jose, CA, USA) equipped with an ESI source (HESI-II probe). The mass spectrometer was operated in positive ESI mode for cyanotoxins and in both modes for STXs. The source temperature was set at 500 °C and capillary temperature at 350 °C. The spray voltage was 4000 V for positive ESI and 3000 V for negative ESI. Air was used as a nebulizing gas with a sheath gas pressure of 60 (arbitrary unit) and an auxiliary gas pressure of 20. The collision gas was argon with a gas pressure of 1.5. The mass spectrometer was operated in selected reaction monitoring (SRM) mode. All SRM transitions and collision energy (CE) are listed in Table 4 for the cyanotoxins and in Supplementary Table S6 for STXs. A mass resolution of 0.7 Da full width at half maximum (FWHD) was set on the first (Q1) and the third (Q3) quadrupoles. Instrument control and data were monitored by a computer equipped with TSQ Tune Master version 2.3.0, Xcalibur version 4.1.31.9 and TraceFinder version 4.1 (Thermo Fisher Scientific, San Jose, CA, USA).

#### 4.5.2. HILIC-HRMS Analysis

The chromatographic conditions used for the analysis of ATXs in HRMS are those described in Table 3. The chromatographic system was conditioned before and after the analytical sequence, as previously described in HILIC-LRMS. The toxins were separated in isocratic mode: 2% mobile phase A and 98% mobile phase B1 for 10 min, at a flow rate of 0.4 mL/min. 

Measurements were carried out on a Dionex Ultimate 3000 HPLC system (Thermo Fisher Scientific, San Jose, CA, USA) coupled with a QTOF (Sciex 5600 Triple TOF, Darmstadt, Germany). The QTOF system was equipped with a DuoSpray ion source and a TurboIonSpray^TM^ probe. For the MS detection, electrospray ionization (ESI) was used in positive mode. The parameters for positive ionization were as follows: ion source gas (GS) 1 and 2, 35 and 45 psi; curtain gas, 30 psi; source temperature, 500 °C; ion spray voltage floating, 5.5 kV; declustering potential, 60 V; ion release delay, 67 ms; ion release width, 25 ms.

The MS was operated in full scan TOF MS and MS/MS modes with information-dependent acquisition (IDA) in a single run analysis for targeted and non-targeted screening. The full scan experiment (50–300 Da) was performed with an accumulation time of 0.1 s while using the high-sensitivity mode. An additional eight MS2 spectra experiments (accumulation time: 0.05 s) were programmed. A collision energy spread (CES ± 20 eV) was applied in conjunction with the CE (40 eV) for IDA mode to perform both low- and high-collision energy, simultaneously resulting in valuable fragmentation information for identification purposes. The mass spectrometer was recalibrated automatically after five measurements while using an automated calibrant delivery system via the atmospheric pressure chemical ionization probe of the DuoSpray ion source.

The presence of ATX analogues was monitored in suspect screening mode via a XIC list in the MasterView^TM^ software (Sciex). The information details about the ATXs monitored are presented in Table 2.

#### 4.5.3. Determination of the Sea Figs Matrix Effects in HILIC-LRMS Analysis

The matrix effect affecting the analysis in HILIC-LRMS of ATX-a in sea figs was determined by comparing calibration curves in solvent (MeCN 82.5% + HAc 0.25%) and in a sea fig extract. The calibration curves included five levels of ATX-a ranging from 0.145 to 4.375 ng/mL.

The sea fig control was used as a matrix and was prepared according to the protocol presented above. The toxin levels of the matrix calibration curve were prepared using this extract. As the control sample naturally contained a small concentration of ATX-a (22.5 µg/kg), the corresponding signal (area) was subtracted at each level of the matrix calibration (cal.) curve, for correction purposes.

The matrix effect was determined using the following equation:

$$\mathrm{Matrix}\;\mathrm{effect}\;\left(\%\right)=\frac{\mathrm{slope}\;\mathrm{cal}{.\;\mathrm{curve}}_{\mathrm{matrix}}-\;\mathrm{slope}\;\mathrm{cal}{.\;\mathrm{curve}}_{\mathrm{solvent}}}{\mathrm{slope}\;\mathrm{cal}{.\;\mathrm{curve}}_{\mathrm{solvent}}}$$

A negative result indicates ion suppression and a positive result, ion enhancement.

## 5. Conclusions

Three of the 18 food poisoning cases reported in France from January 2011 to March 2018 following the consumption of sea figs (*Microcosmus*) were investigated. The HILIC-LCMS analyses in low- and high-resolution unequivocally revealed the presence of ATX-a in concentrations ranging from 193.7 to 1240.2 µg/kg. This is the first time that this freshwater toxin has been reported in sea figs as the possible toxicant responsible for human food poisoning. The range of the ATX-a concentrations found in the sea fig samples was lower than the animal toxicological data reported in the literature. This raises questions about the toxin level necessary to make consumers sick but could also indicate the presence of unknown ATX analogues or the concomitant action of another toxicant.

Further investigations are necessary to explain how a freshwater toxin can be present in marine organisms and to evaluate the hypothesis of the production by a benthic marine cyanobacterium such as *Hydrocoleum* or *Trichodesmium*. Furthermore, it would be interesting to determine if other marine organisms are also contaminated, to study the trophic transfer of ATX-a along the food chain to contribute to the risk assessment and to determine if there might be an ATX-a background level in sea figs explaining why the control sample was contaminated.

## Figures and Tables

**Table 1:** ATX-a concentrations found in the sea fig samples involved in the food poisoning case Numbers 1, 6 and 17, and in the control sample.

Sample	ATX-a Concentration (µg/kg)
FP-1-2011	193.7
FP-6-2012	1240.2
FP-17-2018	1132.6
Sea fig control	22.5
Mussel control	<LOD *

(*) LOD = limit of detection (8 µg/kg).

**Table 2:** Exact mass of the ATX analogues and Phe monitored in suspect screening mode, in HILIC-HRMS on a Sciex 5600 QTOF.

Toxin	Formula	Mass (Da)	Extraction Mass [M + H]^+^ (Da)
ATX-a	C10H15NO	165.11536	166.12264
hATX-a	C11H17NO	179.13101	180.13829
Carboxy ATX-a	C11H15NO3	209.10519	210.11247
Carboxy hATX-a	C12H17NO3	223.12084	224.12812
Carboxy dihydroATX-a	C11H17NO3	211.12084	212.12812
N-methyl ATX a	C11H17NO	179.13101	180.13829
(10*S*)-ATX alcohol	C10H17NO	167.13101	168.13829
(10*R*)-ATX alcohol	C10H17NO	167.13101	168.13829
nor ATX-a	C9H13NO	151.09971	152.10699
Dihydro ATX-a	C10H17NO	167.13101	168.13829
Dihydro hATX-a	C11H19NO	181.14666	182.15394
Epoxy ATX-a	C10H15NO2	181.11028	182.11756
Epoxy hATX-a	C11H17NO2	195.12593	196.13321
ATX-(a)s	C7H17N4O4P	252.09874	253.10602
Phe	C9H11NO2	165.07898	166.08626

**Table 3:** Chromatographic conditions for the analysis of the toxins in HILIC-LCMS.

Heading	Heading
Column	Acquity Glycan BEH Amide column 130 Å 1.7 µm, 2.1 × 150 mm
Pre-column	Acquity Glycan BEH Amide VanGuard Pre-column, 130 Å 1.7 µm, 2.1 × 5 mm
Column temperature	70 °C
Injection volume (µL)	2 µL

**Table 4:** MS parameters for HILIC-LRMS analysis of the cyanotoxins.

Toxin	Transition *	Precursor Ion (*m*/*z*)	Product Ions (*m*/*z*)	Ionization (+/-)	Collision Energy (V)	S-Lens (V)
ATX	Q	166.1	131.1	+	14	53
	q	166.1	105.1	+	16	53
hATX	Q	180.1	145.1	+	14	56
	q	180.1	117.1	+	20	56
CYN	Q	416.2	336.1	+	20	79
	q	416.2	194.1	+	34	79
doCYN	Q	400.1	194.1	+	32	99
	q	400.1	320.1	+	20	99

(*) Q: quantitative transition; q: confirmation transition.

## Supplementary Materials

The following are available online at https://www.mdpi.com/1660-3397/18/6/285/s1: Table S1: Chromatographic conditions for the Conditioning of the HILIC-LCMS system. Table S2: Chromatographic conditions for the Shutdown of the HILIC-LCMS system. Table S3: Chromatographic conditions for the Start-Up of the HILIC-LCMS system. Table S4: Chromatographic conditions for the analysis of the hydrophilic cyanotoxins (ATXs, CYNs) by HILIC-LCMS. Table S5: Chromatographic conditions for the analysis of the STXs by HILIC-LCMS. Table S6: MS parameters for HILIC-LRMS analysis of the STXs in the sea fig samples.
