# Supplementary material for: First Evidence of the Presence of Anatoxin-A in Sea Figs Associated with Human Food Poisonings in France
Source: Mar Drugs. 2020 May 29;18(6):285. doi: 10.3390/md18060285 (PMC7344475; doi:10.3390/md18060285)
Supplement: Supplementary file 1 [file marinedrugs-18-00285-s001.pdf]

marine drugs

MDPI

First Evidence of the Presence of Anatoxin-A in Sea Figs Associated with Human Food Poisonings in France

Ronel Biré, Thomas Bertin, Inès Dom, Vincent Hort, Corinne Schmitt, Jorge Diogène, Rodolphe Lemée, Luc De Haro and Marina Nicolas

Supplementary materials

Table S1. Chromatographic conditions for the Conditioning of the HILIC-LCMS system.

Time (min)	Flow rate (ml/min)	% A	% B1 or B2
0.0	0.10	100	0
1.5	0.20	100	0
3.0	0.30	100	0
4.0	0.35	100	0
30.0	0.35	100	0

Table S2. Chromatographic conditions for the Shutdown of the HILIC-LCMS system.

Time (min)	Flow rate (ml/min)	% C	% D
0.0	0.3	100	0
4.0	0.3	100	0
8.0	0.3	0	100
9.0	0.3	0	100
11.0	0.6	0	100
15.0	0.6	0	100

Table S3. Chromatographic conditions for the Start-Up of the HILIC-LCMS system.

Time (min)	Flow rate (ml/min)	% A	% B1 or B2
0.0	0.3	50	50
4.0	0.3	50	50
6.0	0.5	50	50
15.0	0.5	50	50
16.0	0.5	2	98
17.0	0.4	2	98
17.5	0.4	2	98

Table S4. Chromatographic conditions for the analysis of the hydrophilic cyanotoxins (ATXs, CYNs) by HILIC-LCMS.

**Time (min)**	**Flow rate (ml/min)**	**% A**	**% B2**
0.0	0.4	2	98
5.0	0.4	2	98
11.5	0.4	50	50
13.0	0.5	50	50
13.5	0.5	2	98
14.0	0.8	2	98
14.6	0.8	2	98
15.0	0.4	2	98

Table S5. Chromatographic conditions for the analysis of the STXs by HILIC-LCMS.

**Time (min)**	**Flow rate (ml/min)**	**% A**	**% B1**
0	0.4	2	98
5	0.4	2	98
7.5	0.4	50	50
10.5	0.5	50	50
11	0.5	2	98
11.5	0.8	2	98
12.6	0.8	2	98
13	0.4	2	98

(A) H_2O + 0.015% FA + 0.015% NH_3; (B2) 70% ACN + 0.01% FA

Table S6. MS parameters for HILIC-LRMS analysis of the STXs in the sea fig samples.

Toxin	Transition (m/z) (*)	Ionisation mode	Collision energy (V)	S-lens (V)
**C1**	**474,0 – 351,1**	-	24	100
	474,0 – 121,9	-	28	100
**C2**	**396,0 – 291,1**	+	17	65
	474,0 – 121,9	-	28	100
**C3**	**490,0 –410,1**	-	20	100
	412,0 – 332,1	+	17	65
**C4**	**412,0 – 314,1**	+	17	65
	490,0 – 392,1	-	20	100
**GTX 1**	**410,0 – 367,1**	-	17	100
	410,0 – 349,1	-	21	100
**GTX 4**	**410,0 – 367,1**	-	17	80
	412,1 – 314,1	+	19	80
**GTX 2**	**394,1 – 351,1**	-	17	100
	394,1 – 333,1	-	22	100
**GTX 3**	**394,1 – 333,1**	-	22	100
	396,1 – 298,1	+	17	71
**GTX 5**	**380,0 – 300,0**	+	13	58
	378,0 – 122,0	-	28	90
**GTX 6**	**396,0 – 316 ,1**	+	14	70
	394,0 – 121,9	-	23	80
**dcGTX 2**	**351,0 – 164,0**	-	29	100
	351,0 – 333,1	-	18	100
**dcGTX 3**	**353,0 – 255,0**	+	16	66
	351,0 – 333,1	-	18	100
**dcGTX 1**	**367.0 – 274,1**	-	20	100
	367,0 – 349,1	-	18	100
**dcGTX 4**	**369,0 – 271,0**	+	16	66
	367,0 – 349,1	-	18	90
**STX**	**300,1 – 204,0**	+	22	86
	300,0 – 138,0	+	26	86
**dcSTX**	**257,1 – 126,0**	+	36	84
	257,1 – 222,1	+	17	84
**doSTX**	**241,1 – 60,0**	+	22	84
	241,1 – 206,1	+	19	84
**NEO**	**316,0 – 126,0**	+	24	100
	316,0 – 220,0	+	21	100
**dcNEO**	**273,0 – 126,0**	+	22	86
	273,0 – 110,0	+	36	86

(*) Quantitation transitions are indicated in **bold.**
